# Great influence of geographic isolation on the genetic differentiation of *Myriophyllum spicatum* under a steep environmental gradient

**DOI:** 10.1038/srep15618

**Published:** 2015-10-23

**Authors:** Zhigang Wu, Dan Yu, Zhong Wang, Xing Li, Xinwei Xu

**Affiliations:** 1 National Field Station of Freshwater Ecosystem of Liangzi Lake, College of Life Sciences, Wuhan University, Wuhan 430072, PR China

## Abstract

Understanding how natural processes affect population genetic structures is an important issue in evolutionary biology. One effective method is to assess the relative importance of environmental and geographical factors in the genetic structure of populations. In this study, we examined the spatial genetic variation of thirteen *Myriophyllum spicatum* populations from the Qinghai-Tibetan Plateau (QTP) and adjacent highlands (Yunnan-Guizhou Plateau, YGP) by using microsatellite loci and environmental and geographical factors. Bioclim layers, hydrological properties and elevation were considered as environmental variables and reduced by principal component analysis. The genetic isolation by geographic distance (IBD) was tested by Mantel tests and the relative importance of environmental variables on population genetic differentiation was determined by a partial Mantel test and multiple matrix regression with randomization (MMRR). Two genetic clusters corresponding to the QTP and YGP were identified. Both tests and MMRR revealed a significant and strong correlation between genetic divergence and geographic isolation under the influence of environmental heterogeneity at the overall and finer spatial scales. Our findings suggested the dominant role of geography on the evolution of *M. spicatum* under a steep environmental gradient in the alpine landscape as a result of dispersal limitation and genetic drift.

Understanding how natural processes affect population genetic structures and gene flows is an important issue in evolutionary biology. Environmental variables can influence the movement of gametes, individuals, and groups of individuals among natural populations to affect gene flow patterns^1^, which may have a profound influence on the spatial and temporal distribution of genetic variation, and further, evolutionary progress of natural populations^2^,^3^. Recent advances in molecular ecology and landscape genetics make it feasible to dissect the independent roles of landscape features on population genetic differentiation^4^,^5^,^6^. It can also deepen our understanding of how landscape variables affect dispersal and gene flow, and shape patterns of genetic variation in nature^5^,^7^.

For aquatic plants, given that they live in fragmented islands in terrestrial landscapes, severe genetic differentiation by geographic isolation may be common^8^,^9^, representing an increasing genetic differentiation among populations with increasing geographic distance, as a result of reduced gene flow (isolation by distance, IBD^10^). When ecological characteristics of habitats are dissimilar among natural populations, the probability of successful establishment of immigrants or their offspring may reduce. In this situation, the effective gene flow is reduced as a consequence of local adaptation, resulting in a pattern of isolation by adaptation/environment (IBA or IBE^11^), or monopolization under the influence of founder effects (IBM^4^,^12^). However, relative studies on aquatic plants with incorporating environmental variations in the analysis of population divergence are limited^13^ (but see Zhao *et al.*^14^).

Eurasian watermilfoil (*Myriophyllum spicatum* L.) is native to Europe, Asia and northern Africa^15^ and is recognized as a noxious weed in North America due to its ability to rapidly spread in new habitats^16^. This species occurs in various types of inland water bodies as it is tolerant to a wide range of water and climatic conditions^17^, which may be partly responsible for its cosmopolitan occurrences. Eurasian watermilfoil is widely distributed in China, and previously, we found that both geographic barriers and climate significantly affected population genetic differentiation across the entire country (Wu *et al.* unpublished). In the waters of the Qinghai-Tibetan Plateau (QTP) and adjacent highlands, *M. spicatum* is a common submerged macrophyte^18^. The large range in elevation and the complex landscape of this region offer an ideal place for us to explore the spatial genetic patterns of *M. spicatum* and the underlying factors.

In this study, thirteen Eurasian watermilfoil populations were collected in the QTP and adjacent highlands (Yunnan-Guizhou Plateau, YGP) for genetic analysis using microsatellite markers. The Bioclim layers (climate) and elevation were recruited as environmental variables along with the hydrological properties, which are important for aquatic plants^19^,^20^, for landscape analyses. Our aims were to (1) reveal population genetic structure and gene flow pattern; (2) assess the relative role of geographic isolation and environment heterogeneity in population genetic structure; and (3) find the main force driving genetic divergence of *M. spicatum* in the alpine environment.

## Results

### Genetic diversity and population genetic structure

The number of alleles (NA), the number of effective alleles (NEA) and the genotype diversity (D) ranged from 30 to 75, 25.76 to 50.596 and 0.111 to 1, respectively (Table 1). Globally, the mean NA, NEA and D were 47.846, 38.474 and 0.803, respectively. A total of 102 genets were identified, and all of the obtained genotypes were population specific.

**Figure 1:**
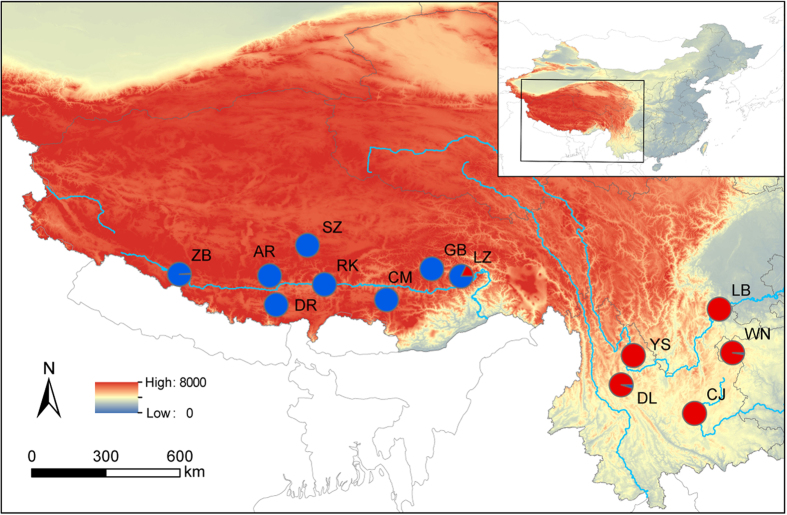
Thirteen sampling sites of *Myriophyllum spicatum* in the QTP and adjacent highlands mapped using ArcGIS. Pie charts represent the probability of assignment to subclusters when K = 2, as identified by STRUCTURE based on microsatellite data. Population codes are shown on the side. The elevation range and main rivers are visualized.

**Figure 2:**
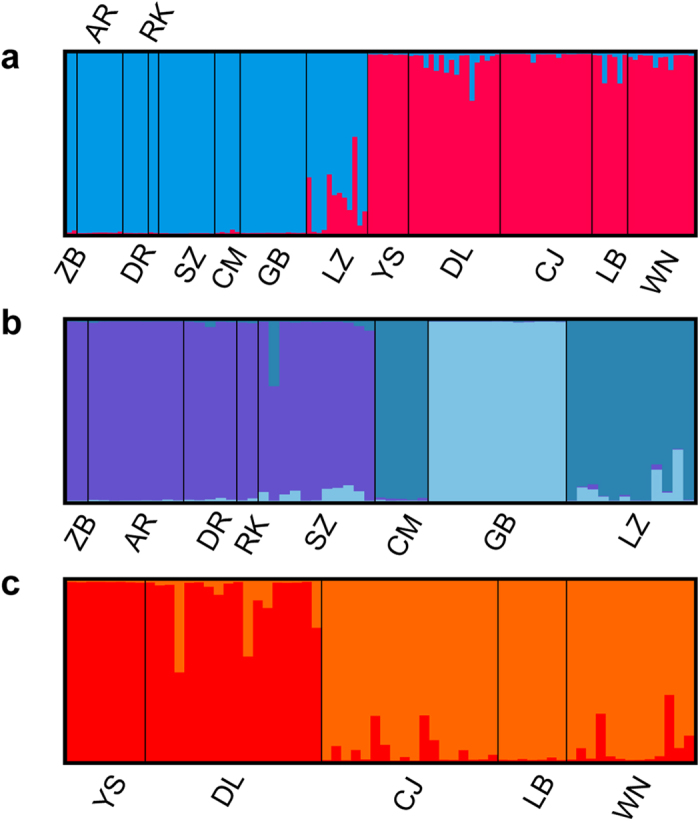
The bar plot depicts the STRUCTURE admixture coefficients of all genets when K = 2 (**a**), the genets of the QTP populations when K = 3 (**b**), and the genets of the YGP populations when K = 2 (**c**). A single vertical bar displays the membership coefficient of each individual, with sample site labeled.

The pairwise Bruvo distance ranged from 0.31 to 0.592, with comparisons involving individuals from AR-DR and DR-DL presenting the greatest and smallest genetic dissimilarity, respectively (Table 2). The STRUCTURE analysis suggested K = 2 as the optimal number of clusters based on the calculation of ∆K (Supplementary Fig. S1). When the studied *M. spicatum* populations were divided into two clusters, we found that the eight populations from the QTP were assigned to one cluster and the five populations of the adjacent highlands (YGP) were assigned to another cluster (Figs 1 and 2a). In separate STRUCTURE analyses for each cluster, obviously internal structure was revealed in both clusters. The QTP cluster was further divided into three subclusters (Supplementary Fig. S1). One of these was comprised five western populations, and the other two subclusters comprised three eastern populations (Fig. 2b). The YGP cluster was further divided into two subclusters (Supplementary Fig. S1). Two western populations were assigned to one subcluser, and three eastern populations were assigned to another subcluster (Fig. 2c). DAPC analyses revealed a pattern consistent with STRUCTURE analyses, with the individuals from the QTP and YGP assigned to distinct clusters without genetic admixture (Supplementary Table S1).

### Isolation by distance

**Figure 3:**
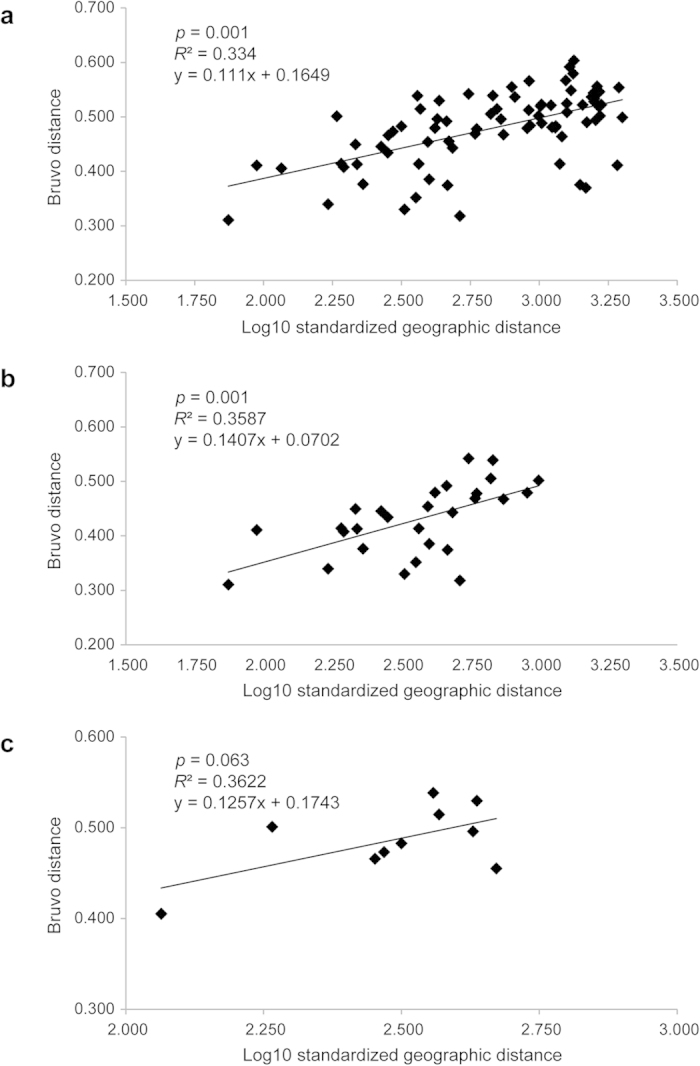
Scatterplots of Mantel tests: (**a**) IBD across the entire data set; (**b**) IBD in the populations of the QTP; (**c**) IBD in the populations of the YGP. The IBD slope is 0.111, 0.141 and 0.126 for entire region, the QTP and the YGP, respectively.

Using Mantel tests, we revealed a significantly positive correlation between geographic and genetic distances (*r* = 0.578, *p* = 0.001, Fig. 3a) at the overall scale. Similarly, a significantly positive correlation was revealed in the QTP cluster (QTP: *r* = 0.559, *p* = 0.001, Fig. 3b). For the YGP cluster, although the correlation was not significant (*r* = 0.602, *p* = 0.063, Fig. 3c), the *p* value of Mantel test was close to 0.05, suggesting that influence of IBD on genetic differentiation were close at the overall and finer scales.

### Landscape genetic analysis

With PCA, we kept the first axis as an environmental variable because it explained 69.3% of the environmental variables, whereas the percentage of inertia explained by other axes of PCA was low (the results of landscape analysis changed little when one or two more axes were included; not shown). For the QTP and YGP clusters, the first two axes explained 69.8% and 80.2% of environmental variables, respectively, which were used to calculate environmental dissimilarity. The relative contributions of environmental variables to the axes are presented in Table S2.

When the influence of the other factor was controlled, the genetic-geographical association (*r* = 0.330, *p* = 0.012) was significant, whereas we could not detect significant correlations between genetic differentiation and environmental distance (*r* = 0.077, *p* = 0.554) (Table 3). With MMRR analysis, the geographic distance had a higher regression coefficient, whereas the effects of environmental factors were not significant (geographic distance: β_D_ = 0.49, *p* = 0.010; environment: β_E_ = 0.108, *p* = 0.559; Table 4). Similarly, in the QTP cluster, both partial Mantel tests and MMRR revealed significant genetic-geographical association (*r* = 0.556, *p* = 0.001; β_D_ = 0.619, *p* = 0.002) and not significant genetic-environmental association (*r* = 0.044, *p* = 0.849; β_E_ = 0.041, *p* = 0.831) (Tables 3 and 4). In the YGP cluster, no significant correlation between genetic distance and geographic distance or environmental distance was found when the influence of the other factor was controlled, but the regression coefficients of genetic-geographical association were about three times higher than those of genetic-environmental association (Tables 3 and 4).

### Discussion

In the present study, a genetic structure was revealed where the QTP and YGP populations were assigned to independent genetic clusters (Fig. 1) corresponding to distinct geographic ranges. Further analyses revealed obviously internal phylogeographic structure in both regions (Fig. 2b,c). A strong association between geographic and genetic distances at different spatial scales (Fig. 3) indicated the pattern of IBD in the alpine populations of *M. spicatum*. The IBD pattern was further confirmed by landscape analyses at the overall and finer spatial scales when the influence of environmental factors was considered (Tables 3 and 4). It suggested that the geographic isolation was the main factor influencing gene flow among populations of *M. spicatum* in the QTP and adjacent highlands.

The IBD pattern was highly associated with limited dispersal among populations in alpine environments. Suren & Ormerod^21^ and Lacoul^22^ suggested that the dispersal of freshwater macrophytes among alpine lakes was limited by the isolation and infrequent visitation of animal vectors. Given few connections by water systems, the inter-population gene flow of *M. spicatum* should rely only on passive dispersal mediated by animals. Waterfowl play important roles in the seed dispersal of many aquatic plants^9^,^23^, and the fact that some waterbirds (e.g., *Anas crecca*) help to transport the seeds of watermilfoils through digestive track^24^,^25^ indicates that the dispersal of Eurasian watermilfoil is likely to be closely linked with waterfowl. However, in China, few waterfowl breed in or migrate across the QTP and YGP regions^26^,^27^. High genetic differentiation and limited gene flow were also detected in some species of aquatic plants in the same region^28^,^29^. Therefore, the single means of dispersal and lack of mediators might aggravate the isolation of *M. spicatum* populations in alpine landscapes, which could explain the greater prediction of IBD on genetic differentiation as a result of dispersal limitation and genetic drift.

A series of environmental factors varied along the elevation gradient, and among them, climate was a fundamental factor that could cause genetic divergence in the population^30^,^31^. Higher-altitude environments contained severe constraints (e.g., short growing seasons and low temperature) on the survival and reproduction of plants and populations located at different altitudes; thus, the divergence might be due to local adaptation^30^,^32^. In the present study, no significant correlation existed between genetic differentiation and the environmental dissimilarity that mainly arose from elevational differences when the effects of geographic isolation were considered, suggesting that the genetic adaptation of *M. spicatum* populations might be limited. Wang *et al.*^33^ found that aquatic plants in the QTP could offset the stress induced by low temperatures and a short growing season via nutrient accumulation and an expedited growth rate, which indicated that physiological adaptation might be important for *M. spicatum* in reacting to alpine environmental stress. In addition, phenological shifts (e.g., flowering period) reduced pollen exchanges between the populations with different elevations and were thus considered to be an important mechanism for creating genetic divergence among populations along the elevational gradient^34^. However, this mechanism should not greatly contribute to genetic divergence in *M. spicatum* populations with different elevations because the inter-population gene flow of this submerged macrophyte mainly relies on vegetative propagules for local propagation and on seeds for long-distance propagation rather than on pollen diffusion^17^.

In conclusion, we highlighted the influence of geographic isolation on the pattern of the gene flow and genetic differentiation of *M. spicatum* under a steep environmental gradient in an alpine landscape. However, physical barriers and climate rather than geographic distance significantly affected the population genetic differentiation of *M. spicatum*, as revealed at the country scale in China (Wu *et al.* unpublished). Different results confirmed that the main factor driving genetic divergence might vary in different regions or at different scales^35^. A variety of studies are needed to help us obtain a deeper understanding of how abiotic factors influence the evolutionary processes of freshwater plants.

## Methods

### Sample collection and DNA extraction

A total of 206 individuals of *M. spicatum* were collected at 13 sites of the QTP and its adjacent highlands in August 2012 (Table 1). We sampled between 6 and 20 plants from each population according to the population size. The plants were collected randomly at intervals of at least 20 m to avoid collecting ramets from a single genet in the clonal Eurasian watermilfoil. Hybridization between *M. spicatum* and its closely related species *M. sibiricum* has been reported in North America^36^,^37^. In our previous study, we confirmed that hybridization between these two species also occurred in the QTP of China^38^. Hybrid populations identified by morphological traits and genetic data were not included in the present study.

Fresh leaves were dried in silica gel in the field and frozen at −20 °C after being transported to the laboratory. Total genomic DNA was extracted using the DNA Secure Plant Kit (Tiangen Biotech, Beijing, China) following the manufacturer’s protocol.

### Genetic diversity and population genetic structure

We previously isolated 20 microsatellite loci from *M. spicatum*^39^. Fourteen of these loci (Myrsp1–7, Myrsp9, Myrsp12–16, and Myrsp18) were used in the present study because of their successful amplification in all populations. The protocols for polymerase chain reaction (PCR) amplification and the analyses of the obtained PCR products followed Wu *et al.*^39^. Genotyping was performed using GeneMapper 4.0 software (Applied Biosystems, Foster City, California, USA).

The indices of genetic diversity, the number of alleles (NA), the number of effective alleles (NEA), the number of genotypes (clone assignment), and Nei’s^40^ diversity index (corrected for sample size) (D) were calculated using GenoType and GenoDive^41^ for each population. Only the genotypes of the genets were kept for subsequent analysis (Table 1).

Because the allelic copies of microsatellites were ambiguous in polyploid species, we could not determine the exact genotypes of heterozygotes in *M. spicatum* due to dosage problems. Additionally, the statistics of microsatellite markers were developed for diploid organisms and were not suitable for polyploid organisms^42^. Therefore, we converted the microsatellite data to a binary format as dominant data^43^,^44^ for genetic structure analysis. In this study, the Bruvo distance (D_B_^42^), based on the shared presence or/and absence of alleles, was recruited for the pairwise individual genetic relatedness. The values were calculated in R software version 3.1.1 (R Development Core Team, 2014) with the package POLYSAT^45^. This index has been proven to be an adequate estimator for polyploids when full genotypes are not required^46^. Because individuals from the same site cannot be considered as independent samples, we randomly picked one individual per sampling site to calculate pairwise Bruvo distances. We repeated the process 100 times and averaged the values of the genetic distance obtained across all of the random sub-samples of individuals for each pair of populations^47^.

Distinct genetic clusters of the 13 Eurasian watermilfoil populations were identified using an individually based assignment approach as implemented in STRUCTURE 2.3.4 48,49, and the current version of the software can accommodate dominant markers^50^. Twenty independent runs were performed for each K value (K = 1 to 10) with a burn-in period of 20,000 iterations and 100,000 MCMC iterations under the admixture model. The best-fit number of clusters was determined based on the ∆K method^51^. The geographical distribution of different clusters identified was mapped using ArcGIS 9.0 (Esri, Redlands, CA, USA). In order to assess potential hierarchical structure, each identified cluster was also analyzed separately.

An alternative method, Discriminant Analysis of Principal Components (DAPC^52^), was also used for the genetic structure. DAPC is an adequate analysis of genetic clustering for polyploidy organisms and does not require the populations to be in Hardy-Weinberg equilibrium^46^. The method required a priori clustering algorithms determined by k-means, and the best number of clusters was assessed using the Bayesian information criterion (BIC)^52^. We evaluated up to k  =  10 groups, but the value of BIC kept decreasing with the increase of k. We therefore set the identical k value of DAPC as the K of STRUCTURE for comparison. DAPC was implemented in R with “adegenet” version 1.4–1^53^.

### Isolation by distance

Mantel tests with 10,000 permutations were used to detect IBD pattern based on matrices of pairwise Bruvo distance and geographic distance among all populations, as implemented in GenAlEx 6.5 software^54^. Because two genetic clusters corresponding to the QTP and YGP were identified (see results), we also performed Mantel tests in each region separately. The geographic distance (log10 standardized) was calculated with the software PASSaGE 2 55 based on the coordinates.

### Landscape genetics analysis

The climatic variables of the studied sites (to a resolution of 1 km) were extracted from the BioClim dataset with GIS information^56^,^57^ using ArcGIS. The properties of the water were also considered. When sampling, the pH, salinity and dissolved oxygen of the aquatic environment were measured using a handheld multiparameter meter (PROPLUS, YSI, USA), and ammonium nitrogen, nitrate nitrogen, the total nitrogen and phosphorus concentrations were determined with a Palintest 7500 Photometer (Palintest, UK).

We reduced the environmental variables by the components of principal component analysis (PCA) based on the 27 environmental variables (including 19 BioClim variables, 7 variables of water conditions and the elevation of the study sites) using the “prcomp” function in R. Prior to analysis, all Bioclim variables and elevation values were standardized.

The correlations between genetic differentiation and geographic/environmental factors were determined by a combination of partial Mantel tests^58^ and matrix regression analysis with a distance matrix. The environmental distance between populations was the Euclidean distance calculated with the values of PCA axes. Partial Mantel tests with 10,000 permutations were performed between genetic factors and one factor under the influence of the other, as implemented in R using the “ecodist” package^59^. Multiple matrix regression with randomization (MMRR) is a novel and robust approach for estimating the independent effects of potential factors, especially in situations of low-to-moderate gene flow^60^. The analysis was implemented with 10,000 permutations in R with the MMRR function script^60^. Because two genetic clusters corresponding to the QTP and YGP were identified (see results), partial Mantel tests and MMRR were also conducted in each region separately.

## Additional Information

**How to cite this article**: Wu, Z. *et al.* Great influence of geographic isolation on the genetic differentiation of *Myriophyllum spicatum* under a steep environmental gradient. *Sci. Rep.* **5**, 15618; doi: 10.1038/srep15618 (2015).

## Supplementary Material

Supplementary Information

## Figures and Tables

**Table 1:** Geographic origins, sample sizes, number of genets, number of alleles (NA), number of effective alleles (NEA), Nei’s genotypic diversity index (D) and the value of the first axis of PCA to the environmental variables (PC1) for the 13 *Myriophyllum spicatum* populations studied.

**Populations**	**Location**	**Latitude**	**Longitude**	**Altitude**	**Population size (ramets)**	**Number of genotypes (genets)**	**NA**	**NEA**	**D**	**PC1**
	QTP									
ZB	Zhongba, Xizang	29.6957	84.1284	4569	14	2	33	32.27	0.264	−2.46
AR	Angren, Xizang	29.2085	87.4154	4357	12	9	44	36.176	0.955	−4.247
DR	Dingri, Xizang	28.5824	87.6523	4253	6	5	45	37.772	0.933	−3.203
SZ	Shenzha, Xizang	30.7542	88.7873	4692	18	2	32	30.226	0.111	−4.069
RK	Rikaze, Xizang	29.3245	89.4102	3787	18	11	45	37.702	0.882	−3.616
CM	Cuomai, Xizang	28.7710	91.6686	4626	18	5	36	33.04	0.81	−4.853
GB	Gongbujiangda, Xizang	29.8927	93.4645	3560	20	13	30	25.76	0.926	−2.357
LZ	Linzhi, Xizang	29.6309	94.3825	2990	12	12	53	43.008	1	−0.12
	YGP									
YS	Yongsheng, Yunnan	26.6237	100.6526	1520	20	8	43	40.166	0.805	4.713
DL	Dali, Yunnan	25.6708	100.2109	1954	19	18	73	50.596	0.994	5.495
CJ	Chengjiang, Yunnan	24.6323	102.8828	1735	20	17	75	49.504	0.984	5.793
LB	Leibo, Sichuan	28.4286	103.7854	1134	16	7	44	35.798	0.775	4.97
WN	Weining, Guizhou	26.8383	104.2645	2187	13	13	69	48.146	1	3.954
Mean							47.846	38.474	0.803	
Total					206	122	136	34.692	0.987	

**Table 2:** Pairwise Bruvo distance between 13 *Myriophyllum spicatum* populations.

	**ZB**	**AR**	**DR**	**SZ**	**RK**	**CM**	**GB**	**LZ**	**YS**	**DL**	**CJ**	**LB**
AR	0.33											
DR	0.414	0.31										
SZ	0.374	0.413	0.445									
RK	0.318	0.407	0.414	0.34								
CM	0.467	0.479	0.454	0.351	0.376							
GB	0.479	0.478	0.469	0.492	0.385	0.449						
LZ	0.502	0.539	0.505	0.542	0.443	0.434	0.411					
YS	0.502	0.603	0.549	0.567	0.483	0.566	0.555	0.515				
DL	0.518	0.579	0.592	0.524	0.48	0.512	0.536	0.496	0.405			
CJ	0.554	0.556	0.528	0.537	0.522	0.464	0.521	0.52	0.483	0.473		
LB	0.411	0.494	0.533	0.37	0.375	0.414	0.523	0.484	0.515	0.455	0.53	
WN	0.499	0.522	0.546	0.544	0.49	0.508	0.481	0.488	0.538	0.496	0.466	0.501

**Table 3:** Partial Mantel tests of association between genetic distances and geographic distances, and the environmental dissimilarity of *Myriophyllum spicatum* populations in the QTP and YGP, and in two study regions respectively.

**Regions**	**Matrix pair**	**Controlled**	***r***	***p***
QTP and YGP	G × Dist	Env	0.330	**0.012**
	G × Env	Dist	0.077	0.554
QTP	G × Dist	Env	0.556	**0.001**
	G × Env	Dist	0.044	0.849
YGP	G × Dist	Env	0.528	0.100
	G × Env	Dist	0.184	0.268

G, genetic distance (Bruvo distance); Dist, geographic distance; Env, environmental distance. Significant values were presented in bold.

**Table 4:** Regression coefficient (β) and significance (*p*) of MMRR analysis on the association between genetic distance and geographic distance, and environmental dissimilarity of *Myriophyllum spicatum* populations in the QTP and YGP, and in two study regions respectively.

**Regions**	**Landscape feature**	**β**	***p***
QTP and YGP	Geographic distance	0.490	**0.010**
	Environmental dissimilarity	0.108	0.559
QTP	Geographic distance	0.619	**0.002**
	Environmental dissimilarity	0.041	0.831
YGP	Geographic distance	0.536	0.186
	Environmental dissimilarity	0.161	0.559

Significant values are presented in bold.
