# Supplementary material for: Great influence of geographic isolation on the genetic differentiation of Myriophyllum spicatum under a steep environmental gradient
Source: Sci Rep. 2015 Oct 23;5:15618. doi: 10.1038/srep15618 (PMC4616052; doi:10.1038/srep15618)
Supplement: Supplementary Information [file srep15618-s1.pdf]

Great influence of geographic isolation on the genetic differentiation of *Myriophyllum spicatum* under a steep environmental gradient

Zhigang Wu, Dan Yu*, Zhong Wang, Xing Li, Xinwei Xu*

National Field Station of Freshwater Ecosystem of Liangzi Lake, College of Life Sciences, Wuhan University, Wuhan 430072, PR China

* Correspondence and requests for materials should be addressed to X. X. (xuxw@whu.edu.cn) and D. Y. (lakeyd@163.com)

Figure S1 Modelling of the number of genetic clusters in the *Myriophyllum spicatum* populations of the whole study regions (a), and of the QTP (b) and the YGP (c) respectively, using STRUCTURE. The magnitude of Δ*K* indicates the most likely number of genetic clusters (left axis, black dots) and the mean (± SD) log probabilities of the data (Ln P(D)) over 20 runs (right axis, open dots and error bars) for each value of *K* were showed.

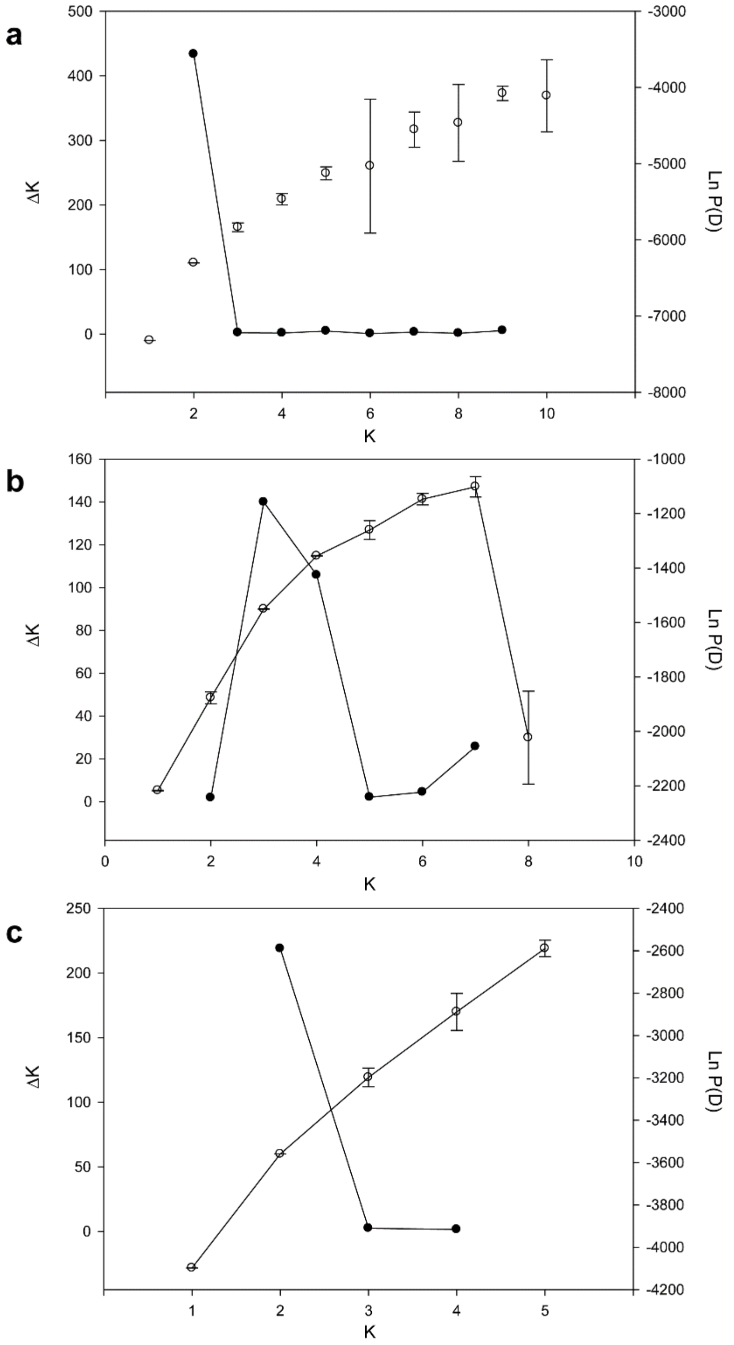

Table S1 Number of genets assigned to two genetic clusters for each population based on the DAPC analysis.

Populations	Number of genotypes (genets)	Number of genets assigned to the QTP group	Number of genets assigned to the YGP group
ZB	2	2	0
AR	9	9	0
DR	5	5	0
SZ	2	2	0
RK	11	11	0
CM	5	5	0
GB	13	13	0
LZ	12	12	0
YS	8	0	8
DL	18	0	18
CJ	17	0	17
LB	7	0	7
WN	13	0	13

Table S2 Contributions of each environmental variable in the dataset of the whole study regions, and of the QTP and YGP respectively, in the PCA analyses.

	QTP and YGP			QTP		YGP	
	PC1 (69.3%)	PC2 (10.4%)	PC3 (9.5%)	PC1 (40.2%)	PC2 (29.6%)	PC1 (53.8%)	PC2 (26.4%)
BIO1 - Annual Mean Temperature	0.198	-0.077	-0.236	-0.207	-0.172	0.248	-0.089
BIO2 - Mean of monthly (max temp - min temp)	-0.206	-0.022	-0.268	0.146	-0.235	0.225	0.179
BIO3 - Isothermality (BIO2/BIO7) * 100	-0.018	0.206	-0.473	-0.133	0.066	0.233	0.153
BIO4 - Temperature Seasonality (standard deviation *100)	-0.201	-0.18	0.18	0.22	-0.207	-0.234	-0.119
BIO5 - Max Temperature of Warmest Month	0.216	-0.218	-0.083	-0.148	-0.305	0.137	-0.249
BIO6 - Min Temperature of Coldeast Month	0.206	-0.01	-0.162	-0.288	0.019	0.261	-0.018
BIO7 - Temperature Annual Range (BIO5-BIO6)	-0.222	-0.152	-0.002	0.2	-0.246	-0.216	-0.068
BIO8 - Mean Temperature of Wettest Quarter	0.219	-0.192	-0.075	-0.18	-0.281	0.182	-0.216
BIO9 - Mean Temperature of Driest Quarter	0.234	-0.065	-0.004	-0.282	0.007	0.261	-0.004
BIO10 - Mean Temperature of Warmest Quarter	0.221	-0.192	-0.073	-0.174	-0.288	0.182	-0.216
BIO11 - Mean Temperature of Coldest Quarter	0.233	-0.081	-0.009	-0.273	-0.084	0.261	-0.004
BIO12 - Annual Precipitation	0.236	0.008	0.019	-0.296	0.018	0.164	-0.195
BIO13 - Precipitation of Wettest Month	0.226	-0.15	-0.051	-0.167	-0.246	0.236	0.04

BIO14 - Precipitation of Driest Month	0.226	0.058	0.104	-0.165	0.206	-0.011	-0.291
BIO15 - Precipitation Seasonality (Coefficient of Variation)	-0.169	-0.361	-0.135	0.191	-0.268	0.144	0.294
BIO16 - Precipitation of Wettest Quarter	0.231	-0.115	-0.021	-0.231	-0.194	0.249	0.048
BIO17 - Precipitation of Driest Quarter	0.203	0.24	0.104	-0.134	0.289	0.061	-0.312
BIO18 - Precipitation of Warmest Quarter	0.228	-0.132	-0.027	-0.213	-0.22	0.249	0.048
BIO19 - Precipitation of Coldest Quarter	0.187	0.292	0.111	-0.12	0.3	0.061	-0.312
PH	-0.155	0.195	-0.338	0.026	0.058	0.174	0.275
Salinity	0.009	-0.212	-0.341	0.127	-0.085	0.104	0.292
Dissolved oxygen	-0.068	-0.163	0.45	0.143	0.017	-0.247	0.07
Ammonium nitrogen content of water	-0.085	-0.304	-0.224	0.045	-0.196	-0.124	-0.237
Nitrate nitrogen content of water	-0.128	-0.348	0.041	0.196	-0.137	-0.226	0.177
Total nitrogen content of water	-0.171	0.221	-0.128	-0.067	0.081	-0.161	0.263
Total phosphorus content of water	0.184	0.216	-0.122	-0.213	0.101	-0.11	-0.023
Elevation	-0.224	0.123	-0.016	0.264	0.161	0.026	0.17
